# Adverse childhood experiences affect the health of middle-aged and older people in China: The multiple mediating roles of sleep duration and life satisfaction

**DOI:** 10.3389/fpsyt.2023.1092971

**Published:** 2023-03-22

**Authors:** Haojie Yin, Xueying Qiu, Yan Zhu, Qing Yang

**Affiliations:** ^1^Geriatrics, Respiratory and Critical Care Department, The First People’s Hospital of Neijiang, Neijiang, China; ^2^Department of Hepatobiliary Surgery, The First People’s Hospital of Neijiang, Neijiang, China; ^3^Sichuan Clinical Research Center for Cancer, Sichuan Cancer Hospital & Institute, Sichuan Cancer Center, Affiliated Cancer Hospital of University of Electronic Science and Technology of China, Chengdu, China

**Keywords:** adverse childhood experiences, depression, activities of daily living, chronic disease, mediation analysis

## Abstract

**Background:**

Although a significant amount of literature has examined the association between childhood adversity and adverse health outcomes, which may be affected by sleep duration and life satisfaction. However, this relationship has not been researched in the Chinese population. This study aimed to assess the association between childhood adversity and health outcomes, with sleep duration and life satisfaction as mediators.

**Methods:**

A total of 14,693 subjects aged 45 and over from the 2018 China Health and Retirement Longitudinal Study (CHARLS) were included. Taking childhood adversity as the independent variable, the health level of middle-aged and older individuals as the dependent variable, and sleep time and satisfaction as the mediating factors, Mplus 8.0 software was used to establish a structural equation model (SEM) to analyze the link between childhood adversity and health level and to explore the mediating effect of target mediators between childhood adversity and health level.

**Results:**

In this study, childhood adversity was positively associated with depression symptoms, activities of daily living (ADL), and the number of chronic diseases (*r* = 0.116, 0.026 and 0.050, respectively, *P* < 0.001). Associations between adverse childhood experiences (ACEs) and depressive symptoms, ADL, and number of chronic diseases were mediated by sleep duration and life satisfaction, respectively.

**Conclusion:**

Adversity experienced in childhood can affect the health status of middle-aged and older people. By ensuring adequate sleep and improving life satisfaction, health outcomes can be improved, and the negative effects of childhood adversity can be reduced.

## Introduction

Childhood is a critical period of growth and development across the life course of individuals, and experiencing adversity during this period can cause significant damage to health, as well as profound and long-lasting negative health consequences (1, 2). Childhood adversity, also known as adverse childhood experiences (ACEs), refers to a series of potentially traumatic experiences that occur before the age of 18. The term ACE is used interchangeably with childhood abuse and childhood trauma. Conditions such as exposure to sexual, physical or emotional abuse and neglect during childhood or living with guardians who have substance abuse problems or psychopathology are manifestations of ACEs (3–5). The definitions of ACE vary from study to study, a typical definition of ACE typically includes harms that affect individuals directly (e.g., child abuse and neglect) and indirectly through negative family or social circumstances (e.g., substance abuse, family conflict, and mental illness) (6). According to a study in the USA (3), more than half of the respondents reported at least one ACE, and more than a quarter of respondents reported two or more ACEs. Another study of the different countries (e.g., UK, Finland, Canada, China, New Zealand, the Philippines, Saudi Arabia, and Sri Lanka), covering different age groups, showed that 57% of the participants reported at least one ACE, and 13% reported at least four ACEs (6). Consequently, there is a high prevalence of childhood adversity worldwide.

Many previous studies have identified ACEs as significantly associated with an increased risk of adverse health outcomes later in life and that the number of adverse experiences is also closely related to the number of adverse outcomes. For example, individuals who have experienced multiple ACEs are more likely to have three or more adverse outcomes than those who have not experienced ACEs (7). At the same time, studies have confirmed that different antecedent risks can often lead to similar outcomes and that these risks are frequently related. Therefore, people who experience one type of ACE are likely to be exposed to multiple ACEs. We conducted a retrospective study of adverse experiences (e.g., abuse, neglect, family dysfunction, and domestic violence) and matched them with health data collected from patients. The results showed that ACEs are associated with an increased risk of many chronic diseases in adulthood, including heart disease, high blood pressure, diabetes, cancer, death, and psychological problems (8, 9). The reason for the above consequences may be that adversity that occurs early in life is so important for changes in brain structure that these changes do not normalize even after later environmental improvements (10). Therefore, the impact of ACEs on adult health is profound, lasting and irreversible. Increasingly, this impact of ACEs has received worldwide attention as a public health problem (11).

It is well known that exposure to violence and abuse in childhood has serious consequences for survivors, including social wellbeing, individual behavior and development, and physical and mental health throughout the lifespan (12–15). In addition to these possible direct consequences, there are indirect ways in which ACEs may have undesirable consequences. Several studies have identified that adolescents and adults who have experienced childhood abuse, neglect, or trauma have higher rates of insomnia or sleep problems than those who have not (16–18). ACEs are associated with difficulty falling asleep (19) and can negatively affect sleep quality later in life (20, 21). In addition, sleep deprivation is considered to be one of the main factors associated with health problems (22, 23). Chen et al. (24) found that decreased sleep quality was associated with an increased risk of depression. Another cohort study from the United States assessed the relationship between ACEs and life satisfaction and showed that people who had experienced ACEs reported significantly lower levels of satisfaction than those who had not and that the more ACEs they experienced, the less satisfied they reported (25). Mersky et al. (7) also confirmed that severe adversity is associated with worse health and lower life satisfaction, which suggests that the association between childhood adversity and health in adulthood may be mediated, at least in part, by certain factors, with sleep and life satisfaction being potential mediators between childhood adversity and health.

This mechanism can be explained by life course theory (26), life course theory is a new interdisciplinary theory proposed by sociologist Elder in the 1960s, the theory explicitly suggests that the life course of individuals is embedded in and shaped by the events they experience over their lifetime. Elder believes that different life events are interrelated, and that events experienced in the early stages of life will affect the occurrence of subsequent life events, which can be used to understand how adversity experiences in childhood are directly or indirectly related to health and wellbeing in adulthood. In the past few decades, the theory of life course has appeared in the epidemiological study of chronic diseases and has a more mature application (27). The theoretical model of life course enables researchers to explicitly test not only the relationship between early life course exposures and later diseases, but also the possible pathways of potential intermediaries or confounding factors (28).

In summary, previous studies had shown that ACEs can reduce sleep quality and life satisfaction, both of which are strongly associated with poor health outcomes. Although there is a large amount of ACE research, the sample representation of previous ACE-related studies in China is limited, and most of the research has focused on the incidence of ACEs and its relationship with health. However, there is little empirical research on how ACEs affect health in adulthood and what mediators affect its influence. Based on the life course theory as the framework theory, this study hypothesizes that ACEs negatively affects sleep duration and life satisfaction, and thus health in adulthood. Sleep duration and life satisfaction mediate the effects of ACEs on adult health. Therefore, the aim of this study is to use representative data from China to assess the relationship between childhood adversity and adult health. The multiple mediating effects of sleep and life satisfaction were also assessed.

## Materials and methods

### Data source

The data used in this study were from the China Health and Retirement Longitudinal Study (CHARLS) in 2018. The CHARLS is a nationally representative longitudinal survey of people aged 45 years or older and their spouses in China. The first nationwide baseline CHARLS data were collected between 2011 and 2012, and respondents were followed every 2 years (29). To ensure the representativeness of the sample, the CHARLS baseline survey covers 150 regions and 450 villages/urban communities across the country, involving 10,257 households, reflecting the overall situation of middle-aged and older people in China. In 2018 was the fourth national survey, covering a wide range of information, including assessments of demographic characteristics, health status, insurance and health utilization, and socioeconomic status of community residents. The life history of the 2014 survey included family history, health history, education history, wealth history and work history of all surviving respondents in the previous two times. According to the research needs, this study adopted the survey data in 2014 and 2018 and matched the data of 19,752 respondents surveyed in 2018 with the life course questionnaire data in 2014 by personal ID. The samples that were surveyed twice were retained, and the effective sample size was 14,693.

China Health and Retirement Longitudinal Study was approved by the Ethics Review Board of Peking University in 2008 (IRB00001052-11015). The methods used in this study were in accordance with the applicable CHARLS specifications and guidelines. All the respondents participated voluntarily and signed the informed consent form. This study was authorized by the CHARLS database.

### Variable description

#### The dependent variable

In 1946, the World Health Organization (WHO) proposed that “health is a state of physical, mental and social integrity, not just the absence of disease and infirmity.” Although many researchers have made different interpretations of the definition of health according to their own research needs, it is only those who reach the state of physical, mental, and cognitive health who are considered to be in a state of health. Considering the comprehensiveness of health measurements, this study chose the number of chronic diseases, activities of daily living (ADL), and depression symptoms to comprehensively reflect the physical and mental health status of middle-aged and older individuals.

Therefore, the dependent variables that this study focuses on are the number of chronic diseases, ADL and depression symptoms of middle-aged and older individuals. First, this study selected questions and answers regarding 14 chronic diseases in the CHARLS questionnaire and counted the number of chronic diseases that respondents suffered from as one of the dependent variables in this paper (30, 31). Second, ADL are designed according to the international general indicator proposed by Katz et al. 1963 (32) and combined with the unique living habits of Chinese people. ADL includes the most basic 11 aspects of daily activities. This study synthesized the answers to daily activities questions from the CHARLS questionnaire. Each question corresponds to four options from “I do not have any difficulty” to “I cannot do it,” and each option is assigned 1, 2, 3, or 4 points from the smallest to the largest. The scores of these 11 questions are added together to obtain the corresponding score, with a total score range of 11–44. A higher total score indicates poorer daily activity performance. In the sample of this study, ADL was shown to have high internal consistency (Cronbach’s alpha = 0.829) and validity (KMO = 0.873, *P* < 0.01). Finally, the Center of Epidemiologic Studies Depression Scale, 10-item version (CES-D) was used to evaluate depression symptoms, which has been widely used in many countries and has been proven to have good reliability and validity in the Chinese population (33). The CES-D scale consists of 10 questions asking respondents to recall how they felt and behaved during the past week, with answers to each question ranging from “rarely or none of the time (<1 day) ‘to’ most or all of the time (5–7 days)” assigned a score of 1–4. The responses to two positive questions in the scale (I felt hopeful about the future; I am happy) were scored in reverse and were assigned a score of 4, 3, 2, and 1, respectively, and the scores were added together to obtain the total score. Therefore, a higher total score indicates a higher severity of depression. In the sample of this study, the CES-D was also shown to have high internal consistency (Cronbach’s alpha = 0.806) and validity (KMO = 0.882, *P* < 0.01).

#### The independent variables

The independent variables in this study are adverse experiences in childhood. Adverse experiences in childhood are divided into three categories: abuse, neglect and household dysfunction, which include physical abuse, emotional abuse, sexual abuse, emotional neglect, physical neglect, parental divorce, domestic violence, bullying, mental abnormality of family members, family crime, and family substance abuse (34). According to our research needs, 24 types of ACEs were selected in this study to form the adverse childhood experiences questionnaire (ACE-Q), including parental divorce, peer bullying, family mental disorder, family substance abuse, family crime, adverse emotional experience, physical abuse, witnessing domestic violence and so on.

Based on a cumulative scoring framework (19), for each of the above adverse experiences, a score of “1” is given to the respondents who have experienced it, and a score of “0” is given to those who have not. The final total score was summed, ranging from 0 to 24, with higher cumulative scores indicating more adverse ACEs (35). In the sample of this study, ACE-Q was shown to have good internal consistency (Cronbach’s alpha = 0.731).

#### Mediating variables and control variables

Sleep duration and satisfaction were mediating variables in this study. Sleep duration was examined by asking respondents: “During the past month, how many hours of actual sleep did you get at night (average hours for one night)? (This may be shorter than the number of hours you spend in bed).” This measure of nighttime sleep duration has been widely used in Chinese studies (36, 37). Sleep duration ranges from 0–24 h. Satisfaction was obtained by asking respondents, “Thinking about life as a whole, are you satisfied with it?” The answers to this question are assigned 1–5 points from “completely satisfied” to “not at all satisfied,” respectively, and higher satisfaction scores indicate lower life satisfaction. This one-dimensional measure of life satisfaction is commonly used in large-scale or nationwide surveys in China (38, 39).

The control variables of this study use the most basic demographic characteristics variables, including a continuous variable of age and two binary variables of marital status (reference group: married) and gender (reference group: female). Additionally, other studies have shown that health levels in childhood correlate with health outcomes in adulthood (30). Therefore, this study also included childhood health history as a control variable in the model.

Childhood health history was obtained by asking the respondents: “Before you were 15 years old (including 15 years old), would you say that compared to other children of the same age, you were much healthier, somewhat healthier, about average, somewhat less healthy or much less healthy?” A “much healthier, somewhat healthier or about average” response was defined as “0” healthy, otherwise “1” unhealthy.

#### Statistical analysis

**FIGURE 1:**
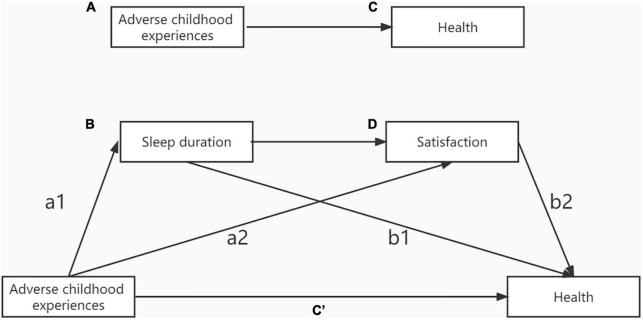
**(A)** The direct relationship between adverse childhood experiences and **(C)** health levels. **(B)** Sleep and **(D)** satisfaction mediate the relationship between adverse childhood experiences and health.

First, IBM SPSS version 25.0 was used for descriptive statistics and correlation analysis, and a multiple imputation method was used to impute the missing data in this study. Descriptive statistics included the percentage of categorical variables and the mean and standard deviation of continuous variables in the entire sample. For continuous variables, the *t*-test is used for the date conforming to the normal distribution, and the Kruskal-Wallis test is used for the data not conforming to normal distribution. Next, the structural equation model (SEM) was established using Mplus 8.0 software to further examine the effect of ACEs on health, as shown in Figure 1. This study hypothesized that ACEs had a predictive effect on health in adulthood (H1). Sleep duration (H2) and satisfaction (H3) mediated the relationship between ACEs and health. Sleep duration and satisfaction may be mediators of the relationship between ACEs and health (H4).

According to the mediation effect test procedure proposed by Professor Wen et al. (40), this paper adopted a three-step method for testing. The first step is to use a completely direct model to analyze the impact of ACEs on the health of middle-aged and older people after controlling for age, sex, marital status, and childhood health history. In the second step, two mediating variables, sleep duration and satisfaction, were added to the model for path analysis. Third, under the condition that the model fit index was acceptable, the maximum likelihood method and Bootstrap were used to test the mediating model, sampling was repeated 5,000 times, and the 95% CI for bias correction was calculated.

Finally, Subgroup analysis was used to determine if standardized regression coefficients (β) differed by subgroup. Gender was classified as female and male. Childhood health history was classified as healthy and unhealthy. Marital Status was classified as married and unmarried. Age was classified as middle-aged (age 60 years or younger), and older (age 60 years or older). The statistical test was significant at the level of *P* < 0.05, according to the significance level.

## Results

### Participant characteristics

As shown in Tables 1, 2. Among the 14,693 samples, 6,854 (46.65%) were male, and 7,839 (53.35%) were female. The average age of the respondents was 61 (SD = 9.64), and married respondents accounted for 86.66% of all participants. Regarding childhood health history, 87.74% of the respondents considered that they were in a healthy state during childhood. Furthermore, the average depression symptoms score of the respondents was 18.71 (SD = 6.59), the average ADL score was 12.49 (SD = 3.33), and 6,447 (43.88%) of the respondents suffered from at least one chronic disease. Nearly 88.51% of the respondents experienced at least one adversity as a child. Respondents slept an average of 6.2 h per night (SD = 1.95), and the average satisfaction score was 2.75 (SD = 0.80). In addition, Table 2 shows that CES-D scores, ADL, sleep duration and satisfaction differed in relation to middle-aged and older people subgroup. CES-D, ADL, sleep duration and satisfaction significantly differed in all subgroup analyses. For chronic disease, subgroup analyses of gender showed few statistically significant differences. ACEs significantly differed in all subgroup analyses except that for marital status.

**TABLE 1:** Socio-demographic characteristics of participants.

Variables	*N*	%
**Gender**		
Female	7,839	53.35
Male	6,854	46.65
**Age**		
45∼60	7,101	48.32
60∼75	6,375	43.39
75∼90	1,207	8.21
>90	10	0.07
**Marital status**		
Married	12,733	86.66
Unmarried	1,960	13.34
**Childhood health history**		
Healthy	12,892	87.74
Unhealthy	1,801	12.26

**TABLE 2:** CES-D, ADL, chronic disease, ACEs, sleep duration, and satisfaction among middle-aged and older people subgroup.

Variables	All (*N* = 14,693)	Gender		*P*	Health history		*P*	Marital status		*P*	Age		*P*
		Female (*N* = 7,839)	Male (*N* = 6,854)		Healthy (*N* = 12,892)	Unhealthy (*N* = 1,801)		Married (*N* = 12,733)	Unmarried (*N* = 1,960)		≤60 (*N* = 7,101)	>60 (*N* = 7,592)	
CES-D	18.71 (6.59)	19.8 (6.93)	17.47 (5.95)	***	18.46 (6.50)	20.55 (6.60)	***	18.40 (6.43)	20.73 (7.26)	***	18.31 (6.36)	19.09 (6.78)	***
ADL	12.49 (3.33)	12.66 (3.42)	12.30 (3.22)	***	12.43 (3.26)	12.99 (3.81)	***	12.36 (3.17)	13.37 (4.14)	***	11.91 (2.35)	13.04 (3.97)	***
Chronic disease	0.82 (1.23)	0.82 (1.23)	0.81 (1.23)	0.59	0.80 (1.22)	0.94 (1.31)	***	0.80 (1.20)	0.94 (1.39)	***	0.70 (1.12)	0.92 (1.31)	***
ACEs	4.49 (2.98)	4.21 (2.96)	4.81 (2.98)	***	4.39 (2.92)	5.22 (3.30)	***	4.50 (2.98)	4.43 (3.01)	0.34	4.69 (2.99)	4.31 (2.96)	***
SD	6.20 (1.95)	6.02 (2.06)	66.41 (1.79)	***	6.24 (1.94)	5.91 (1.98)	***	6.26 (1.90)	5.82 (2.22)	***	6.34 (1.72)	6.08 (2.13)	***
Satisfaction	2.75 (0.80)	2.77 (0.83)	2.72 (0.76)	**	2.73 (0.79)	2.88 (0.85)	***	2.74 (0.78)	2.80 (0.90)	*	2.78 (0.79)	2.71 (0.80)	***
Age	61.17 (9.64)	60.60 (9.73)	61.83 (9.49)	***	61.20 (9.64)	60.98 (9.65)	0.38	60.05 (9.07)	68.46 (10.08)	***	–	–	–

ADL, activities of daily living; CES-D, center of epidemiologic studies depression scale; ACEs, adverse childhood experiences; SD, sleep duration.

***P < 0.001.

**P < 0.01.

*P < 0.05.

### Correlation analysis

Correlation analysis was conducted on the CES-D score, ADL score, number of chronic diseases, ACEs, sleep duration and satisfaction score, and the results showed that the correlation between these key study variables all reached a significant level. ACEs and life satisfaction scores were positively correlated with CES-D scores, ADL scores and the number of chronic diseases. This suggested that higher scores of ACEs and life satisfaction are correlated with greater severity of depressive symptoms, worse activity and more chronic diseases. However, sleep duration was negatively correlated with the CES-D score, ADL score, number of chronic diseases, and ACEs and life satisfaction scores. Detailed results are shown in Table 3.

**TABLE 3:** Correlation coefficients between key study variables (*N* = 14,693).

Variables	CES-D	ADL	Chronic disease	ACEs	Sleep duration	Satisfaction
CES-D	1					
ADL	0.312***	1				
Chronic disease	0.186***	0.176***	1			
ACEs	0.116***	0.026**	0.050***	1		
SD	-0.271***	-0.120***	-0.095***	-0.049***	1	
Satisfaction	0.390***	0.130***	0.094***	0.118***	-0.110***	1

CES-D, center of epidemiologic studies depression scale; ADL, activities of daily living; ACEs, adversity childhood experiences; SD, sleep duration.

***P < 0.001.

**P < 0.01.

### Structural equation model

First, prior to the analysis of the intermediary effects model, we used a total direct model to derive the effects of ACEs on the health of middle-aged and older people. As shown in Table 4, in the model, we include age, gender, marital status, and childhood health history as control variables. The model showed an acceptable fit index: χ2/df = 8.02, CFI = 0.994, TLI = 0.987, RMSEA = 0.022, SRMR = 0.009. The results revealed that ACEs significantly and directly promoted depression symptoms (β = 0.143, *P* < 0.001), ADL (β = 0.048, *P* < 0.001), and chronic diseases (β = 0.056, *P* < 0.001).

**TABLE 4:** Direct model regression weight.

DV	IV	Std. est.	S.E.	Est./S.E.	*P*	*R* ^2^
CES-D	ACEs	0.143	0.009	16.015	***	0.083
	Age	0.070	0.009	7.758	***	
	Gender	-0.198	0.008	-23.419	***	
	Marital	0.090	0.010	9.107	***	
	Healthy history	0.095	0.009	10.407	***	
ADL	ACEs	0.048	0.009	5.418	***	0.064
	Age	0.225	0.010	22.677	***	
	Gender	-0.071	0.009	-8.103	***	
	Marital	0.036	0.011	3.293	**	
	Healthy history	0.055	0.010	5.715	***	
Chronic disease	ACEs	0.056	0.009	6.575	***	0.014
	Age	0.098	0.009	11.323	***	
	Gender	-0.013	0.008	-1.584	0.113	
	Marital	0.010	0.010	1.000	0.317	
	Healthy history	0.031	0.009	3.591	***	

CES-D, center of epidemiologic studies depression scale; ADL, activities of daily living; ACEs, adversity childhood experiences.

***P < 0.001.

**P < 0.01.

### Multiple indirect effects model

**FIGURE 2:**
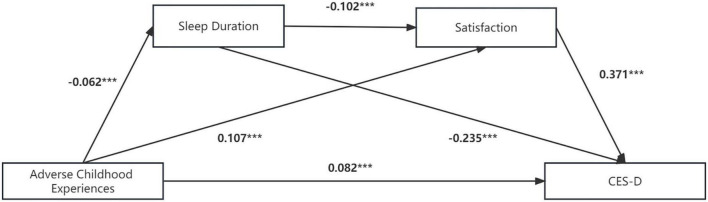
Multiple mediation model between adverse childhood experiences and CES-D scores with sleep duration and life satisfaction as mediators. Path values are the path coefficients. CES-D, Center of epidemiologic studies depression scale. ***P < 0.001.

Second, we established a multiple intermediary effects model to examine the effects of ACEs on depression symptoms, ADL, and chronic diseases through mediators, namely, sleep duration and satisfaction. Similarly, we controlled for age, sex, marital status and childhood health history in this model. Figure 2 and Table 5 show the detailed impact path of ACEs on depression symptoms, and the fit index of the multiple mediation model is acceptable: χ2/df = 64.1, CFI = 0.978, TLI = 0.905, RMSEA = 0.066, SRMR = 0.014. Table 6 shows the total effect, direct effect, indirect effect and their 95% confidence interval (CI), in which the effect value of ACEs on depression symptoms through sleep duration is 0.014, accounting for 10.5% of the total effect. Furthermore, the effect value of ACEs on depression symptoms through life satisfaction was 0.040, accounting for 28.8% of the total effect. The effect value of ACEs on depression symptoms through sleep time and life satisfaction was 0.002, accounting for 1.7% of the total effect. The size of the direct effect of ACEs on depression symptoms was 0.082, accounting for 59.1% of the total effect. The CIs corresponding to each mediation path did not include 0, indicating that the multiple mediating effects between ACEs and depressive symptoms were valid (sleep duration and satisfaction were used as mediating variables). Moreover, the mediating effect of life satisfaction was stronger than that of sleep duration (β = −0.006, *P* < 0.001).

**TABLE 5:** Model regression weight of the impact of ACEs on CES-D scores.

DV	IV	Std. est.	S.E.	Est./S.E.	*P*	*R* ^2^
CES-D	ACEs	0.082	0.008	10.110	***	0.284
	SD	-0.235	0.009	-27.097	***	
	Satisfaction	0.371	0.009	43.356	***	
Satisfaction	ACEs	0.107	0.009	12.270	***	0.032
	SD	-0.102	0.009	-11.342	***	
SD	ACEs	-0.062	0.009	-7.178	***	0.026

DV, dependent variable; IV, independent variable; CES-D, center of epidemiologic studies depression scale; ACEs, adversity childhood experiences; SD, sleep duration.

***P < 0.001.

**TABLE 6:** Total, direct, and indirect effects of ACEs on CES-D, sleep duration and life satisfaction as mediators.

	Point estimate	Product of coefficients			95% CI	
					Bias corrected	
		S.E.	Est./S.E.	*P*	Lower	Upper
**Indirect effects**						
SD	0.014	0.002	6.917	***	0.010	0.019
Satisfaction	0.040	0.003	11.688	***	0.033	0.047
SD→Satisfaction	0.002	0.000	5.910	***	0.002	0.003
Total indirect	0.057	0.004	13.781	***	0.049	0.065
**Direct and total effects**						
Direct	0.082	0.008	10.110	***	0.066	0.098
Total	0.138	0.009	15.447	***	0.121	0.156
**Contrasts**						
SD vs. Satisfaction	-0.006	0.001	-6.266	***	-0.008	-0.004
SD vs SD→Satisfaction	0.003	0.000	6.711	***	0.002	0.004
Satisfaction vs. SD→Satisfaction	0.008	0.001	10.984	***	0.007	0.010

SD, sleep duration.

***P < 0.001.

**FIGURE 3:**
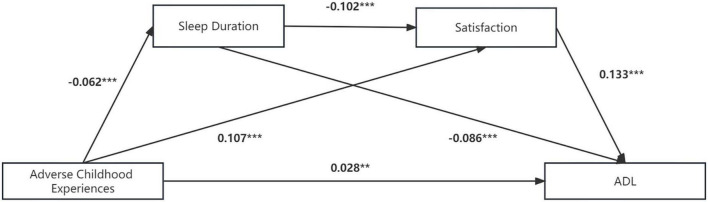
Multiple mediation model between adverse childhood experiences and ADL with sleep duration and satisfaction as mediators. Path values are the path coefficients. ADL, Activities of daily living. ***P < 0.001 and **P < 0.01.

Figure 3 and Table 7 show the detailed impact path of ACEs on ADL, and the fit index of the multiple mediation model is acceptable: χ2/df = 5.8, CFI = 0.997, TLI = 0.993, RMSEA = 0.018, SRMR = 0.006. Table 8 shows the total effect, direct effect, indirect effect and their 95% CI, in which the effect value of ACEs on ADL through sleep duration is 0.005, accounting for 10.9% of the total effect. Furthermore, the effect value of ACEs on ADL through satisfaction was 0.014, accounting for 29.6% of the total effect. The effect value of ACEs on ADL through sleep time and satisfaction was 0.0001, accounting for 1.7% of the total effect. The size of the direct effect of ACEs on ADL was 0.028, accounting for 57.8% of the total effect. The CIs corresponding to each mediation path did not include 0, indicating that the multiple mediating effects between ACEs and ADL were valid (sleep duration and life satisfaction were used as mediating variables). Moreover, the mediating effect of life satisfaction was stronger than that of sleep duration (β = −0.001, *P* < 0.001).

**TABLE 7:** Model regression weight of the impact of ACEs on ADL.

DV	IV	Std. est	S.E.	Est./S.E.	*P*	*R* ^2^
ADL	ACE	0.028	0.009	3.203	**	0.091
	SD	-0.086	0.012	-7.250	***	
	Satisfaction	0.133	0.011	12.536	***	
Satisfaction	ACE	0.107	0.009	12.270	***	0.032
	SD	-0.102	0.009	-11.342	***	
SD	ACE	-0.062	0.009	-7.178	***	0.026

DV, dependent variable; IV, independent variable; ADL, activities of daily living; ACEs, adversity childhood experiences; SD, sleep duration.

***P < 0.001.

**P < 0.01.

**TABLE 8:** Total, direct, and indirect effects of ACEs on ADL, sleep duration and satisfaction as mediators.

	Point estimate	Product of coefficients			95% CI	
					Bias corrected	
		S.E.	Est./S.E.	*P*	Lower	Upper
**Indirect effects**						
SD	0.005	0.001	4.939	***	0.003	0.008
Satisfaction	0.014	0.002	8.625	***	0.011	0.018
SD→Satisfaction	0.001	0.000	5.335	***	0.001	0.001
Total indirect	0.020	0.002	10.237	***	0.017	0.024
**Direct and total effects**						
Direct	0.028	0.009	3.203	**	0.011	0.045
Total	0.048	0.009	5.456	***	0.031	0.065
**Contrasts**						
SD vs. Satisfaction	-0.001	0.000	-4.398	***	-0.001	-0.001
SD vs. SD→Satisfaction	0.000	0.000	4.438	***	0.000	0.001
Satisfaction vs. SD→Satisfaction	0.001	0.000	8.130	***	0.001	0.002

SD, sleep duration.

***P < 0.001.

**P < 0.01.

**FIGURE 4:**
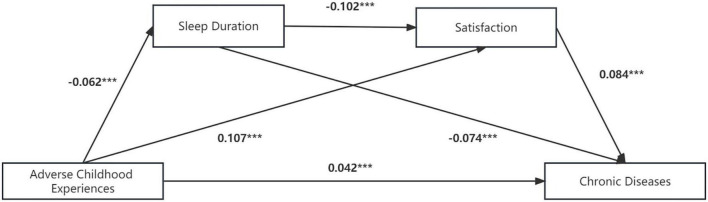
Multiple mediation model between adverse childhood experiences and chronic diseases with sleep duration and life satisfaction as mediators. Path values are the path coefficients. ***P < 0.001.

Figure 4 and Table 9 show the detailed impact pathways of ACEs on chronic diseases, and the multiple mediation model fits perfectly: χ2/df = 0, CFI = 1.000, TLI = 1.000, RMSEA = 0.000, SRMR = 0.000. Table 10 shows the total effect, direct effect, indirect effect and their 95% CI, in which the effect value of ACEs on chronic diseases through sleep duration is 0.005, accounting for 8.1% of the total effect. Furthermore, the effect value of ACEs on chronic diseases through life satisfaction was 0.009, accounting for 16.1% of the total effect. The effect value of ACEs on chronic diseases through sleep time and satisfaction was 0.001, accounting for 0.9% of the total effect. The size of the direct effect of ACEs on chronic diseases was 0.42, accounting for 74.8% of the total effect. The CIs corresponding to each mediation path did not include 0, indicating that the multiple mediating effects between ACEs and chronic diseases were valid (sleep duration and life satisfaction were used as mediating variables). Moreover, the mediating effect of life satisfaction was stronger than that of sleep duration (β = −0.002, *P* < 0.01).

**TABLE 9:** Model regression weight of the impact of ACEs on the number of chronic diseases.

DV	IV	Std. est	S.E.	Est./S.E.	*P*	*R* ^2^
Chronic diseases	SD	-0.074	0.009	-8.250	***	0.027
	Satisfaction	0.084	0.009	9.254	***	
	ACEs	0.042	0.009	4.835	***	
Satisfaction	ACEs	0.107	0.009	12.271	***	0.032
	SD	-0.102	0.009	-11.342	***	
SD	ACEs	-0.062	0.009	-7.178	***	0.026

DV, dependent variable; IV, independent variable; ACEs, adversity childhood experiences; SD, sleep duration.

***P < 0.001.

**TABLE 10:** Total, direct, and indirect effects of ACEs on chronic diseases, sleep duration and satisfaction as mediators.

	Point estimate	Product of coefficients			95% CI	
					Bias corrected	
		S.E.	Est./S.E.	*P*	Lower	Upper
**Indirect effects**						
SD	0.005	0.001	5.391	***	0.003	0.006
Satisfaction	0.009	0.001	7.249	***	0.007	0.012
SD→Satisfaction	0.001	0.000	4.975	***	0.000	0.001
Total indirect	0.014	0.002	9.082	***	0.011	0.017
**Direct and total effects**						
Direct	0.042	0.009	4.835	***	0.025	0.059
Total	0.056	0.009	6.427	***	0.039	0.074
**Contrasts**						
SD vs. Satisfaction	-0.002	0.001	-2.925	**	-0.003	-0.001
SD vs SD→Satisfaction	0.002	0.000	5.039	***	0.001	0.002
Satisfaction vs. SD→Satisfaction	0.004	0.001	6.994	***	0.003	0.005

SD, sleep duration.

***P < 0.001.

**P < 0.01.

### Subgroup analyses

The results of subgroup analyses are shown in Table 11. The mediating effect of sleep duration was significant in multiple subgroups, for example, in the female group (β = 0.016, *P* < 0.001), in male (β = 0.012, *P* < 0.001), in healthy group (β = 0.016, *P* < 0.001), in unmarried (β = 0.028, *P* < 0.001). The mediating effect of satisfaction was significant in all subgroups. The effect values of ACEs on depression symptoms, ADL, and chronic diseases through sleep time and satisfaction were significant in multiple subgroups except for people in unhealthy group (β = 0.001, *P* > 0.05). In multiple subgroups, the direct effect of ACEs on ADL was not statistically significant except in the female group (β = 0.033, *P* < 0.01), the healthy group (β = 0.026, *P* < 0.01), the married group (β = 0.025, *P* < 0.01), and the older group (age over 60 years) (β = 0.039, *P* < 0.01), this suggests that the effect of ACEs on ADL was through complete mediation in most subgroups. Furthermore, analyses of additional indices in each subgroup indicated that the models were appropriate (e.g., in female group, χ2/df = 35.72, CFI = 0.982, TLI = 0.919, RMSEA = 0.067, SRMR = 0.014).

**TABLE 11:** Standardized regression coefficients (β) with *P*-values for the components of subgroup analyses.

Path	Gender		Health history		Marital status		Age	
	Female (*N* = 7,839)	Male (*N* = 6,854)	Healthy (*N* = 12,892)	Unhealthy (*N* = 1,801)	Married (*N* = 12,733)	Unmarried (*N* = 1,960)	≤60 (*N* = 7,101)	>60 (*N* = 7,592)
ACEs *via* SD to CES-D	0.016***	0.012***	0.016***	0.005 (0.365)	0.012***	0.028***	0.012***	0.017***
ACEs *via* satisfaction to CES-D	0.040***	0.040***	0.038***	0.050***	0.041***	0.030**	0.044***	0.036***
ACEs *via* SD and satisfaction to CES-D	0.003***	0.002***	0.003***	0.001 (0.380)	0.002***	0.005**	0.002**	0.003***
ACEs to CES-D	0.069***	0.102***	0.086***	0.053*	0.085***	0.054*	0.070***	0.090***
ACEs *via* SD to ADL	0.007***	0.003*	0.005***	0.004 (0.373)	0.004**	0.015**	0.003*	0.007***
ACEs *via* satisfaction to ADL	0.014***	0.014***	0.014***	0.018***	0.014***	0.012**	0.014***	0.015***
ACEs *via* SD and satisfaction to ADL	0.001***	0.001**	0.001***	0.000 (0.393)	0.001***	0.002**	0.001**	0.001***
ACEs to ADL	0.033**	0.021 (0.081)	0.026**	0.041 (0.074)	0.025**	0.045 (0.080)	0.010 (0.440)	0.039**
ACEs *via* SD to chronic disease	0.005***	0.004**	0.005***	0.001 (0.431)	0.004***	0.010**	0.004**	0.005***
ACEs *via* satisfaction to chronic disease	0.009***	0.009***	0.009***	0.011**	0.009***	0.007*	0.010***	0.008***
ACEs *via* SD and satisfaction to chronic disease	0.001***	0.000**	0.001***	0.000 (0.410)	0.000***	0.001*	0.000**	0.001***
ACEs to chronic disease	0.043***	0.041**	0.042***	0.039 (0.0397)	0.040***	0.048 (0.051)	0.032*	0.050***

CES-D, center of epidemiologic studies depression scale; ACEs, adversity childhood experiences; ADL, activities of daily living; SD, sleep duration.

***P < 0.001.

**P < 0.01.

*P < 0.05.

## Discussion

To the best of our knowledge, this study is the first to assess in detail the relationship between childhood adversity and health levels in a representative Chinese population aged 45 years and older. In this study, exposure to ACEs was associated with depression symptoms, poor activity in daily life, and multiple chronic diseases among middle-aged and older individuals in China. We constructed three multivariate mediation models to analyze the association mechanism between ACEs and the health level of middle-aged and older individuals. The results showed that ACEs had significant direct effects on depression symptoms, ADL, and the number of chronic diseases in middle-aged and older adults, which supported Hypothesis 1. This is consistent with the study of Heim, Mikaela, Jack P. et al., and the reason may be related to the continuous sensitization of childhood trauma and stress response and the dynamic changes of the hypothalamic axis, which, in turn, is associated with depression symptoms (41). Additionally, early experience of adversity affects physical health in adulthood through accumulation and biological embedding of adversity during developmentally sensitive periods (42), and children who have experienced adversity are more likely to adopt unhealthy lifestyles, which further leads to poorer physical and activity function in adulthood (43). In addition, exposure to adverse experiences in childhood may cause structural changes in different brain regions that are associated with later learning, coping, and stress management (44). Therefore, children who have experienced adverse ACEs are more likely to adopt unhealthy lifestyles as coping mechanisms, such as heavy smoking, alcohol abuse, and sleep disorder (6), which are well-established risk factors for physical and mental health (45). In conclusion, the negative effects of childhood adversity are prevalent and long-lasting and need our attention.

In addition, after adding two mediating variables of sleep duration and life satisfaction, we found that the mediating path was significant, which supported Hypothesis 2, Hypothesis 3, and Hypothesis 4. This indicates that the effects of ACEs on depression symptoms, ADL and the number of chronic diseases can be explained by mediating variables.

First, as we predicted, sleep duration regulates the pathway from ACEs to depression, ADL, and the number of chronic diseases, which has been confirmed by many studies. Several studies have also found that children exposed to ACEs had altered sleep status early in life. Moreover, an assessment of the association between ACEs and sleep disorders in adults found that ACEs were significantly associated with frequent sleep deprivation (17), and sleep played an important role in mediating the association between ACEs and a variety of health outcomes, including immune dysfunction and psychological factors. This provides further evidence that sleep is the key link between ACEs and poorer health (21). Therefore, this suggests that improving sleep quality and reducing the incidence of sleep disturbances may not only reduce the negative effects of childhood adversity but may also improve health later in life.

Second, our results show that ACEs are positively associated with a decline in health through a decrease in life satisfaction, which is consistent with several recent studies. The study of Hughes et al. (46) found that early adversity was negatively correlated with life satisfaction, and the OR value of low life satisfaction increased with the number of ACEs, while low satisfaction was significantly correlated with an increase in the number of chronic diseases per year (47). One possible explanation for the above results is that life satisfaction is a type of cognition and evaluation of subjective wellbeing, which generates positive emotions. Such positive emotions not only encourage people to choose a more active lifestyle (48) but also motivate people to take care of themselves (49), thus promoting individual health. However, abuse and some other stressors experienced in childhood can affect brain development (50) and have negative and long-lasting effects on emotional function (1); thus, people with more ACEs are more likely to rarely or never experience positive emotions (e.g., optimism, relaxation, self-confidence) and feelings of closeness to others. As a result, they have a lower happiness index and a lower level of life satisfaction. These findings provide guidance for health professionals that they can improve health outcomes by enhancing the life satisfaction of individuals with ACEs.

Moreover, subgroup analysis showed that ACEs directly or indirectly influenced depressive symptoms, ADL, and the number of chronic diseases. This effect does not change with gender, marital status, age, and childhood healthy status. This suggests that prevention efforts against poor health outcomes need to shift focus, including early drivers of poor health. The United Nations 2030 Agenda for Sustainable Development shows that countries have committed to take action to achieve the 17 global Sustainable Development Goals by 2030. Importantly, the Sustainable Development Goals also focus on early childhood development as a means of ensuring health throughout life (51), this provides strong support for healthcare workers and policymakers to focus on early ACEs.

### Strengths and limitations

A strength of this study is the use of a large sample covering different regions of China to explore the associations between ACEs and health outcomes in middle-aged and older people. Furthermore, this is not a single health outcome but includes mental, physical and daily living ability, which made the results convincing. This study found that sleep duration and life satisfaction were mediators between ACEs and depressive symptoms, ADL and chronic diseases. For middle-aged and older adults with or without adverse ACEs, the results of this study may provide methods to improve health outcomes from different perspectives.

The limitations of the study must be taken into account when interpreting the results. First, respondents were asked to self-report some ACEs, which means that some memory bias must exist. Memories of people who have had ACEs may be suppressed or amplified, resulting in reporting bias. Second, health outcomes are produced by the joint action of multiple factors, and those that were not included in the study may also have a certain impact on health outcomes. Third, the data used in this study are derived from a cross-sectional survey; although the hypotheses of this study are supported by theory, experience, and statistical data, we are still unable to draw causal inferences from the results. Finally, it is not clear from this study whether different types of ACEs have different effects on health outcomes. Therefore, further studies can explore the relationship between sleep duration, satisfaction, and health level longitudinally, and the specific impact pathways can also be explored in the effects of different types of ACEs on health level *via* a serial mediation of sleep duration and satisfaction. At the same time, we can include more influencing factors in future research to obtain more comprehensive conclusions.

## Conclusion

Despite certain limitations, this study identified the mediating effects of sleep duration and life satisfaction between ACEs and health levels. These results enrich the literature on ACEs and health levels and have certain theoretical implications. Similarly, these results also have clinical implications, which inspire researchers and health professionals to pay as much attention as possible to the ACE of middle-aged and older people in future work. By improving sleep quality and mobilizing positive emotions, the health of populations with ACEs can be enhanced and health outcomes can be improved, thereby reducing the negative effects of childhood adversity.

## Data availability statement

The original contributions presented in this study are included in the article/Supplementary material, further inquiries can be directed to the corresponding author.

## Ethics statement

The studies involving human participants were reviewed and approved by Ethics Review Board of Peking University in 2008 (IRB00001052-11015). The patients/participants provided their written informed consent to participate in this study.

## Author contributions

QY provided guidance on the thinking and writing of the whole text. HY processed part of the data and wrote the full text. XQ carried out the data processing and chart drawing of the empirical part. YZ provided us with the full text of the embellishment. All authors listed meet the authorship criteria according to the latest guidelines of the International Committee of Medical Journal Editors, read, and approved the final manuscript.
